# Classification and Explanation for Intrusion Detection System Based on Ensemble Trees and SHAP Method

**DOI:** 10.3390/s22031154

**Published:** 2022-02-03

**Authors:** Thi-Thu-Huong Le, Haeyoung Kim, Hyoeun Kang, Howon Kim

**Affiliations:** 1 IoT Research Center, Pusan National University, Busan 609735, Korea; lehuong7885@gmail.com; 2 Faculty of Information Technology, Hung Yen University of Technology and Education, Hung Yen 160000, Vietnam; 3 School of Computer Science and Engineering, Pusan National University, Busan 609735, Korea; haeyoung@islab.re.kr (H.K.); hyoeun405@gmail.com (H.K.)

**Keywords:** decision tree, ensemble trees, explanation AI (XAI), intrusion detection systems (IDS), random forest, SHapley Additive exPlanations (SHAP)

## Abstract

In recent years, many methods for intrusion detection systems (IDS) have been designed and developed in the research community, which have achieved a perfect detection rate using IDS datasets. Deep neural networks (DNNs) are representative examples applied widely in IDS. However, DNN models are becoming increasingly complex in model architectures with high resource computing in hardware requirements. In addition, it is difficult for humans to obtain explanations behind the decisions made by these DNN models using large IoT-based IDS datasets. Many proposed IDS methods have not been applied in practical deployments, because of the lack of explanation given to cybersecurity experts, to support them in terms of optimizing their decisions according to the judgments of the IDS models. This paper aims to enhance the attack detection performance of IDS with big IoT-based IDS datasets as well as provide explanations of machine learning (ML) model predictions. The proposed ML-based IDS method is based on the ensemble trees approach, including decision tree (DT) and random forest (RF) classifiers which do not require high computing resources for training models. In addition, two big datasets are used for the experimental evaluation of the proposed method, NF-BoT-IoT-v2, and NF-ToN-IoT-v2 (new versions of the original BoT-IoT and ToN-IoT datasets), through the feature set of the net flow meter. In addition, the IoTDS20 dataset is used for experiments. Furthermore, the SHapley additive exPlanations (SHAP) is applied to the eXplainable AI (XAI) methodology to explain and interpret the classification decisions of DT and RF models; this is not only effective in interpreting the final decision of the ensemble tree approach but also supports cybersecurity experts in quickly optimizing and evaluating the correctness of their judgments based on the explanations of the results.

## 1. Introduction

The intrusion detection system (IDS) has a vital role to play against cyberattacks in global business enterprises and governments. Therefore, both the research and industry communities have been engaged in rapidly developing IDSs. The amount of resources spent annually to fight cybercrime is increasing annually [1]. Denial of services (DoS), web-based attacks, and malicious insiders are the most harmful types of cybercrimes; intellectual properties can be lost because of these attacks. Several businesses or governments are deploying antivirus software, firewalls, and IDS to fight against cybercrime as well as to reduce the annual cost of these attacks. Hence, to provide a more secure network environment, an IDS has become a necessary tool in computer networks. Moreover, the objectives of IDSs are to detect unauthorized use and misuse in the host network [2,3].

Recently, the Internet of Things (IoT) has become attractive in the research community and industry owing to its benefits. IoTs comprise interconnected devices that are embedded with computational tools, such as processing units or sensors. These computational tools support collecting, storing, and exchanging data over the Internet [4]. Although the IoT ecosystem has great potential advantages in automated intelligence and digital capabilities, securing IoT networks has become the main challenge to their implementation or deployment [5]. The main reason for the less secure current state of IoT networks is that criminals can hack into IoT devices when the IoT connects the physical and digital worlds [6]. Therefore, IoT security becomes extremely necessary with the continuous development of internet technology today.

IoT-based IDS has become a popular and essential approach because the number of IoT devices and IoT infrastructure development has increased sharply with the rapid development of wireless networking. Research has pointed out that machine learning (ML) and software-defined networking (SDN)-based IDS are useful tools for fast responses to different attacks in IoT networks [7]. Nevertheless, the ML-based IDS so far has been performed only on old datasets; in other words, these IDS have become out-of-date and are not suitable for modern attacks. Spadaccino and Cuomo [8] analyzed the opportunities and challenges for edge computing in an IDS-based IoT environment. ML was applied to their IDS, which can be leveraged to detect abnormalities. They analyzed the advantages and disadvantages of the IDS application with requirements for real-time response, storage capacity, and computational power.

Traditional IDSs based on ML models have been developed, with most of them having been developed as black-box models with promising detection results on certain IDS datasets. On the one hand, traditional IDS models that use sophisticated algorithms do not provide insights into their behavior and reasoning. In particular, many IDS methods have been designed and developed in the research community, which have achieved a perfect detection rate using IDS datasets in recent years. DNNs are representative examples applied widely in IDS. However, DNN models are becoming increasingly complex in model architectures with high resource computing and hardware requirements. The complexity of ML-based IDS models makes it difficult to explain the reason behind the predictions made. In addition, it is difficult for humans to obtain explanations behind the decisions made by these DNN models using large IoT-based IDS datasets.

Indeed, many proposed IDS methods have not been applied in practical deployments, because of the lack of explanation given to cybersecurity experts to support them in terms of optimizing their decisions according to the judgments of the IDS models. Therefore, experts are reluctant to trust decisions made by IDSs based on ML models [9]. The major influencing factor in trusting any IDS detection is understanding the impact of malicious data on the detection of any intrusion in the system. Most previous studies have concentrated more on the accuracy of various classification models than on placing trust in the IDS. On the other hand, XAI has recently become increasingly important for interpreting ML models. This technique enhances trust in IDS management by allowing human experts to understand the underlying data evidence and causal reasoning of IDS-based ML decisions. Hence, this paper focuses on both aspects: improving IDS model accuracy and explaining the decisions so that it is possible to trust the IDS model.

In this study, two effective ensemble trees are used, namely: DT and RF models for IoT-IDSs datasets to improve the detection rate of attack types. Although these models are conventional models in ML, they are widely applied to both traditional IDSs and other fields. This approach is both efficient and accurate; moreover, it has low complexity and high resource computing requirements compared to advanced DNNs. In addition, this study explains the prediction results of these models using the SHAP technique for local and global explanations. Therefore, the main contributions of this study are as follows.

- Improvement of attack detection performance of IDS with big IoT-based IDS datasets based on ensemble tree models. In particular, in the first step, model selection has performed to determine the best model for tuning, along with adjusted hyperparameters, on preprocessing IoT-based IDS datasets. Better model results and their hyperparameters were used to build the best IDS model. The best IDS model selection algorithm is presented in Algorithm 1; the Pycaret library [10] was used to support the implementation of this algorithm. In the next step, the selected model classification has built with the training and evaluation process by tuning the hyperparameter values. These models are ensemble tree classifications, including DT (decision tree) and RF (random forest). They have been evaluated using the receiver operating characteristic (ROC) curve and validation curve on three IoT-based IDS datasets, including IoTID20, NF-BoT-IoT-v2, and NF-ToN-IoT-v2. The training and evaluation process of the best selected models with turned hyperparameters is presented in Algorithm 2.
- Explanation about attack detection results achieved from a decision of the proposed ensemble IDS methods based on the SHAP (SHapley additive exPlanations) method. The SHAP method [11,12] has been used for the global and local explanation of ensemble trees for binary classifiers and multiclass classifiers to increase the trustability of the prediction results. The SHAP value is calculated based on Algorithm 3. In particular, a heatmap for global explanation and a decision plot for a local explanation was used in the SHAP method. The proposed model explanation with SHAP is presented in Algorithm 4.

The remainder of this paper is organized as follows. Section 2 describes related works, such as the AI-based IDS approach, AI-based IDS-related IoT approach, AI-based IDS for the IoT, and the explanation approach. The related material and proposed method are introduced in Section 3. Section 4 presents the results of the two experiments, including classification model performance evaluation, and explanation of model classification decision with global and local interpretation. Section 5 discusses and compares the proposed method with other methods in the same field. Finally, Section 6 presents the conclusions of the study.

## 2. Related Work

In this section, a review of several learning-based approaches used in traditional IDSs, as well as IDSs related to IoTs, and current IDSs related to IoTs and explanation methods, is presented.

### 2.1. AI-Based IDS Approach

Earlier, various ML models were used for the IDS approach, such as the support vector machine (SVM), naive Bayes, DT, RF, and neural networks. DT and RF might be better approachable than SVM and naive Bayes models. In [13,14,15], the SVM classification detected a set of hyperplanes as separators in a high-dimensional space. However, the difficulty and challenge in this IDS-based SVM are finding a suitable kernel function and the high time complexity of the learning phase. By contrast, Bayesian classifiers [16,17,18] predicted the output classes using Bayes’ rule. In addition, association rules are used to detect normal or abnormal traffic. In the naive Bayesian model, conditionally independent features are assumed. Although this assumption is not valid in practice, experiments have demonstrated its good performance.

A DT is used in IDS with the main data mining technique. To improve signature-based IDS, Kruegel et al. [19] used the DT model; their research indicated that the speed of the DT detection process is significantly improved by experimental results. Kumar et al. [20] applied a decision tree algorithm to detect misuse and anomaly attacks. The proposed method can detect unknown attacks. Moreover, [21] introduced an IDS based on a DT using big data in a fog environment. The proposed method was able to completely detect four types of attacks and enabled the detection of twenty-two other kinds of attacks. Another study [22] combined a DT with sensitive pruning to tackle the privacy issue by modifying the C4.8 decision tree on the 6% GureKDDCup NIDS dataset.

Another ensemble model of DT is RF, which has good performance compared with SVM and neural networks. In particular, the research conducted in [23] applied an RF classifier with information gain-based feature selection that was able to obtain better performance accuracy on the NSL-KDD dataset. The authors of [24] also used the RF model on the NSL-KDD dataset with a high detection rate and low false alarm rate results. Aung et al. [25] proposed a hybrid IDS method based on k-means and RF algorithms on the KDD’99 dataset, with the results indicating a high-performance accuracy and low model training time. Another study [26] used the RF model and adapted it to the Apache Spark distributed processing system to realize real-time detection with satisfactory efficiency and accuracy compared to existing systems. In recent years, researchers [27,28,29] applied random forest to the problem, and their experimental results showed the improved performance of the algorithm compared with the existing algorithms. In addition, Le et al. [30] used the RF model to classify DoS attacks in wireless sensor networks (WSNs). In summary, the RF model can run efficiently on large datasets and features. In addition, this model is robust against overfitting and can handle unbalanced data.

DL methods such as DNN are considered potential methods for IDS in [31,32]. In particular, Recurrent Neural Network (RNN), Long-Short Term Memory (LSTM), and Gated Recurrent Unit (GRU) methods have recently been widely used in IDSs. These networks are suitable for sequential inputs that depend on each other. Le et al. [33] applied an RNN model with a variant effective for activation functions in an IDS. However, this method cannot detect U2R attacks. Next, Kim et al. [34] proposed an LSTM model for an IDS using the KDD cup dataset. Although it obtained a high result of 98.88% for the detection rate (DR), the false alarm rate (FAR) was still high at 10.04%. Hence, the study [35] improved the performance of LSTM on the same dataset, KDD cup, by applying variant gradient descent optimization. The obtained results were 98.95% for RD and 9.98% for FAR. In addition, although the type attack of the U2R classification result was improved by 50% compared to [33], the U2R classification result still needed improvement. Hence, the authors of [36] applied the GRU model with the PCA scale to improve the classification of each attack type, especially the U2R attack type, as well as reduce the FAR value. The accuracy of U2R was 86% with PCA-MinMax and GRU. In addition, the FAR obtained was 1.4% with the same method and KDD cup dataset. In addition, [37] used variant RNN, LSTM, and GRU with a novel feature section model to build robust IDS methods on two datasets: NSL-KDD and ISCX datasets. The results of this study show that the proposed method (SFSDT+GRU) can obtain the best performance among variant RNN models with a detection rate of 91.8% and 90% for detecting U2R attack types. On the other hand, Y. Mirsky et al. [38] proposed the Kitsune method which is a plug and play NIDS (network IDS), based on neural network, and can learn to detect attacks on the local network. The core of Kitsune is KitNET, which is an online algorithm that is efficient enough to run on a single core of a Raspberry PI. However, the authors only tested or evaluated their method on their private dataset. Furthermore, M. Roopak et al. [39] proposed a hybrid method based on Convolution Neural Network (CNN) and LSTM for classifying attacks using the CISIDS2017 dataset with DDoS attacks in IoT Networks. This method was obtained 99.03% in terms of accurate measurement. Nevertheless, their proposed method needs extending to apply and evaluate different cyber-attacks in the IoT environment.

In summary, DNN models have good effective intrusion detection along with attack types classification with high performance. However, DNN-based IDS approaches often used some kinds of traffic flow feature, for example, start time, IP address, destination port, duration time, byte number packets, and packet. These features are generally important for detecting several attacks, including DDoS and portscan. However, almost all IDS datasets contain data with imbalanced outputs, which in turn, reduce the accuracy performance of the variant RNN models because overfitting or underfitting occurs. Another reason is that the requirement for a deep learning model with big data processing requires considerable performance computing and more complexity using a graphical processing unit (GPU) or tensor processing unit (TPU).

### 2.2. AI-Based IDS Related IoT Approach

There are several well-known datasets in IDS, such as ISCX2012 [40], UNSWNB15 [41], and CICIDS2017 [42]; however, these datasets were not collected from an IoT environment. In recent years, several studies have started to focus on IDS with IoT environments, such as NSL-KDD and DS2OS. However, the number of IoT devices and novel attack techniques has grown in recent years. Hence, it is necessary to upgrade datasets to reflect the IoT environment and novel attacks. In addition, the available IoT-based IDS datasets lack a large number of features. Therefore, recent datasets have been introduced, such as the original BoT-IoT [43] and ToN-IoT datasets [44] and their various versions by other authors, such as NetFlow V1 [45] (NF-BoT-IoT-v1 and NF-ToN-IoT-v1) and NetFlow V2 [46] (NF-BoT-IoT-v2 and NF-ToN-IoT-v2) and IoTID20 [47]. These datasets focus more on daily home usage devices, whereas other datasets concentrate on academic network traffic. Consequently, this study considered these datasets to investigate IoT IDS for IoT environments.

#### 2.2.1. IoT-IDS with BoT-IoT, ToN-IoT Datasets and Their Variant Datasets

There are some representative papers that used the BoT-IoT dataset in the proposed methods. For example, D. Oreški and D. Andročec [48] proposed a useful predictive IDS model based on the genetic algorithm which was applied to optimize the neural network (NN) parameters. This method can solve the issue of spending a lot of time in the trial-and-error phase of the traditional NN model process. Although they presented a good performance in terms of F1 measurement, they did experiments to evaluate their method on only public dataset Bot-IoT along with binary classification (normal and attack prediction). Next, [49] proposed a temporal convolution neural network (TCNN) and evaluated it on the BoT-IoT dataset. The research obtained high accuracy for multiclass traffic detection, but TCNN needed to be combined with an oversampling method, SMOTE-NC (synthetic minority oversampling technique-nominal continuous) to solve unbalanced datasets. Therefore, the TCNN model might become more complex in architecture and computation. G. Boventzi et al. [50] proposed H2ID (hierarchical hybrid for intrusion detection) with high performance in both anomaly detection and recognizing unknown attacks tasks, applied on the BoT-IoT dataset. Nevertheless, the research needs to be expanded to evaluate attack types with different thresholds of new datasets.

Recently, Nimbalkar and Kshirsagar [51] proposed an IDS method using features such as information gain (IG) and gain ratio (GR) with JRipclassifier and then evaluated and validated the results on IoT-BoT and KDD Cup 99 datasets, respectively. The unique features with the top 50% ranked feature of the total number of features in the compact dataset were extracted using IG and GR. IG-TFP-FS and GR-TFP-FS have two unique features. Two groups of unique features to obtain sub-features, namely RFS-1 and RFS-2, are generated using intersection and union operations. The JRip rule-based classifier uses these subsets of features to select a single feature subset that includes the minimum number of features. Although the proposed method can obtain high-performance accuracy, their proposed approach might contain complex steps.

On the other hand, the DT model with the J48 algorithm was applied to detect four different IoT attack types in a smart home environment by Anthi et al. [52]. Although the proposed method obtained high precision and recall results as well as a low classification time, there are two existing problems, including using default model parameters for different models and failure to address the class imbalance issue. Therefore, some researchers have improved the performance of their detection IDS methods by optimizing the ML models. For example, [53] combined the Bayesian optimization Gaussian process (BO-GP) algorithm and the DT model in an effective way to detect attacks on IoT devices. The BoT-IoT-2018 dataset was used to evaluate the proposed method and the experimental results of the optimized framework show effectiveness and robustness for detecting botnet attacks in IoT environments. However, the drawback of this method is that it does not use a complete dataset. In other words, the authors reduced the dataset from 72 million records to 3.6 million records, representing 5% of the dataset size used. This results in less enrichment in the normal traces scenario. Another limitation of this research is that it does not consider time-related features to identify temporal behaviors or patterns that may be helpful in detecting botnet attacks in IoT environments.

On the ToN-IoT dataset, [54] discovered six ML models, including a deep feedforward (DFF), convolutional neural network (CNN), RNN, DT, logistic regression (LR), and Naive Bayes (NB), and evaluated them on the ToN-IoT dataset. They concluded that the ML model achieved the best scores for all the datasets used. However, it is very difficult to compare the performance of ML-based IDS classifiers in different network scenarios because of the lack of a standard performance and feature set among the various IDS datasets. This problem was addressed by providing the NetFlow version of two IoT-based IDS datasets [45]. In particular, they used the raw packet capture (pcap) files of the original datasets and then converted to the NetFlow format based on nprobe tool. After that, they extracted 12 features.

Next, Lo et al. [55] proposed E-GraphSAGE for IDS using both versions, such as BoT-IoT & ToN-IoT and NF-BoT- IoT & NF-ToN-IoT compared with extra tree and random forest classifiers in [45]. Another study [56] introduced two approaches, deep feed-forward, and random forest, on both original ToN-IoT and NF-ToN-IoT datasets, and the results showed that the accuracy initially increased rapidly with the addition of features but converged quickly to the maximum achievable detection accuracy.

#### 2.2.2. IoT-IDS with IoTID20 Dataset

In [57], the authors proposed a hybrid method between unsupervised learning and supervised learning. The proposed method includes a clustering with three stages: reduction stage, oversampling stage, and classification by single hidden layer feedforward neural network (SLFN) on the IoTDS20 dataset. Although the obtained result has high accuracy, the proposed architecture is very complex with a high computational cost. Alkahtani et al. [58] proposed a hybrid convolutional neural network with a long short-term memory (CNN-LSTM) model on the IoTDS20 dataset with 98.80% accuracy. However, their approach requires a particle swarm optimization (PSO) algorithm for selecting subset features. Islam et al. [59] identified various types of IoT threats using shallow models, such as DT, RF, and SVM, and deep models such as DNN, deep belief network (DBN), LSTM, stacked LSTM, bidirectional LSTM (Bi-LSTM)) based IDS in the IoT environment. Five benchmark datasets, NSL-KDD, IoTDevNet, DS2OS, IoTID20, and IoT botnet datasets were used to evaluate these models. Next, Song et al. [60] used autoencoders to develop an IDS method. The proposed method used three benchmark datasets, including NSL-KDD, IoTID20, and N-BaIoT. Although the proposed method obtained high performance detection for unknown attack types, this model spent much time and effort to optimize the hyperparameters setting to find the best detection performance. However, recently, Hussein et al. [61] proposed a method using the random forest to combine one-hot encoding for IoT IDS on the IoTID20 dataset. The achieved results were a binary label accuracy of 99.9%, a division label accuracy of 99.3%, and a subcategory label accuracy of 95.8%.

To that end, in this paper, different optimization approaches have been used to build the best performance model with hyper-parameters selected by the model selection process on complete IoT-related IDS datasets, along with using the time-related feature in each dataset used. In addition, Spaqddicino & Cuomo [8] reviewed ML techniques applied to IDS and their main requirements with a high level for input knowledge and training resource requirements, and a low level for memory and operation resource requirements. Hence, this study used base-supervised ML models to develop the proposed methods. In addition, in this study, machine learning methods were applied to the IoTDS20 dataset and the two newest versions of ToN-IoT-v2 and BoT-IoT-v2.

### 2.3. AI-Based IDS for the IoT and Explanation Approach

The authors of [62] mentioned interpretabbility using XAI-based techniques to understand the behavior of ML and DL models. However, humans encounter difficulty in interpreting the decision results of complex ML or DL models. Moreover, XAI mostly focused on popular fields, such as computer vision, biologies, and nature language processing, meanwhile the cybersecurity field was rarely applied. Hence, cybersecurity experts cannot optimize their decision following the judgments of ML or DL models. Explanations give measurable factors as to what features influence the prediction of a cyber-attack and to what degree. Hence, we used a well-known ML-based XAI method, SHAP to provide explanation of our proposed IDS classifiers. The SHAP method was provided in global and local explanation in this work. While global explanation provides important features, the local explanation explains why the model makes certain decision on a specific input. In addition, we provided full interpretation in both cases: binary classification and multi-class classification.

The role of explaining the IDS method’s decision gives measurable factors, such as which feature influences the prediction of a cyber-attack and degree. Marino et al. [63] introduced an explanation interface to provide explanations for misclassified samples. Experimental evaluation was conducted on the NSL-KDD99 benchmark dataset using linear and multilayer perceptron classifiers. Mane & Rao [64] proposed the XAI framework to explain measurable factors as to what features influence the prediction of a cyberattack and the degree to which it is generated from SHAP, local interpretable model-agnostic explanations (LIME), contrastive explanations method (CEM), ProtoDash, and Boolean decision rules via column generation (BRCG). These approaches were applied to the NSL-KDD dataset for intrusion detection systems (IDS) and demonstrated the results. Next, Wang et al. [65] introduced an explainable ML framework for an IDS, where the NSL-KDD dataset was used to test the feasibility of the proposed framework. Mahbooba et al. [66] used XAI to enhance trust management in IDSs using a DT on the KDD dataset and a DT algorithm for decision making.

Moreover, Szczepanksi & Choras [67] introduced a hybrid oracle-explainer approach for IDS, which is a solution that allows for the possible model performance and delivers human-understandable interpretations well. They evaluated their proposed method using the CICIDS2017 dataset. In addition, in two recent research papers [68,69], Sarhan et al. proposed the IDS method as well as the explainable AI method, SHAP, to explain and interpret the classification decisions of two ML models on one of the feature sets of NetFlow, including BoT-IoT and ToN-IoT. Their experimental results show that the NetFlow feature set enhances the detection accuracy of the two ML models.

## 3. Material and Method

This section provides briefly background knowledge related to the proposed method. In particular, the main information of ensemble tree classification, including DT and RF classifiers, SHAP explanation with global and local explanation information, and how to estimate the SHAP value is summarized.

### 3.1. Ensemble Trees Classification

Firstly, the concept of DT involves determining the most informative feature and splitting the data value of these features to become the target features as node representations. The most informative features are searched until they end up with pure leaf nodes. To measure the most informative feature values, this study used the information gain (IG) to measure how the uncertainty of the target variable given by a set of independent variables was reduced. Furthermore, the hyperparameter of DT model is considered because of its effectiveness in the DT model’s performance accuracy in a practical model. Hence, we consider and adjust DT’s parameters comprising [criterion, splitter, max depth, min samples split, min samples leaf, max features, random state, max leaf nodes, max leaf nodes, min impurity decrease, min impurity split, class weight, ccp alpha].

Secondly, an RF is an ensemble tree that is built from a DT model. The forest is created by training an RF using the bagging or bootstrap aggregating technique. The RF is established based on the predictions of the DTs. The final prediction of RF is based on achieving the average or mean output value from various DTs. Similar to the DT model, most of the hyperparameters of the DT model are pointed out, which are in the RF model. Other important hyperparameters include n estimators, min weight fraction leaf, bootstrap, oob score that also are considered to adjust in the practical RF model.

### 3.2. SHAP Explanation

Shapley values, a game-theoretic approach, are often used for optimal credit allocation. SHAP is a method to explain individual predictions and that was proposed by Lundberge and Lee [11]. Furthermore, a variant of SHAP, such as tree-based SHAP, named TreeSHAP was introduced by Lundberg et al. [12]. TreeSHAP has faster performance than KernelSHAP. There are two types of SHAP explanations including global explanation and local explanation. In particular, detailed information on the two kinds of SHAP explanations are presented as follows.

#### 3.2.1. Shap Global Explanation

**Figure 1:**
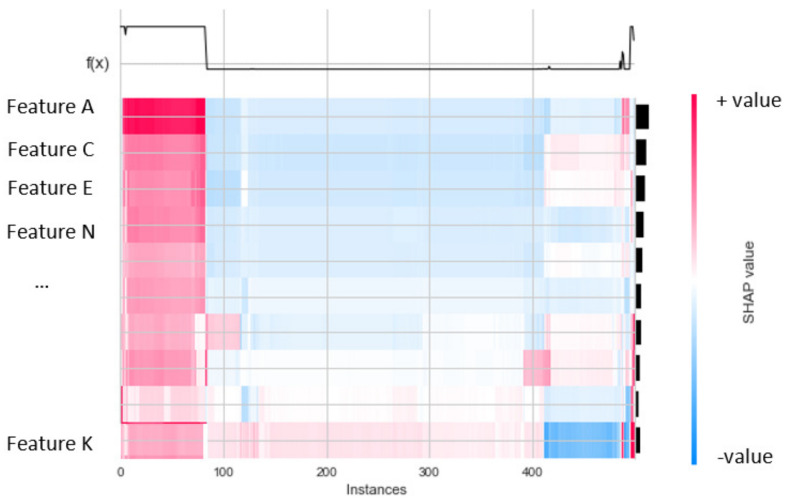
Example of heatmap plot.

In terms of general XAI, global explanation is associated to the average behavior of the method after some pooling/aggregation. The benefit of the global explanation using SHAP values which can show how much each predictor/feature contributes to the output features for either positive or negative values. In this study, a heatmap plot was used to visualize the results of the global explanation of the proposed ML model prediction. The heatmap matrix is used to present the model’s output. Besides, a bar plot on the right-hand side (black color) presents the global importance of each model input (feature). Figure 1 shows an example heatmap plot with features (predictors) and f(x) prediction results. The heatmap above shows high predictions (high values in f(x) to the left) associated with high feature A content (red color) (high SHAP value).

#### 3.2.2. SHAP Local Explanation

**Figure 2:**
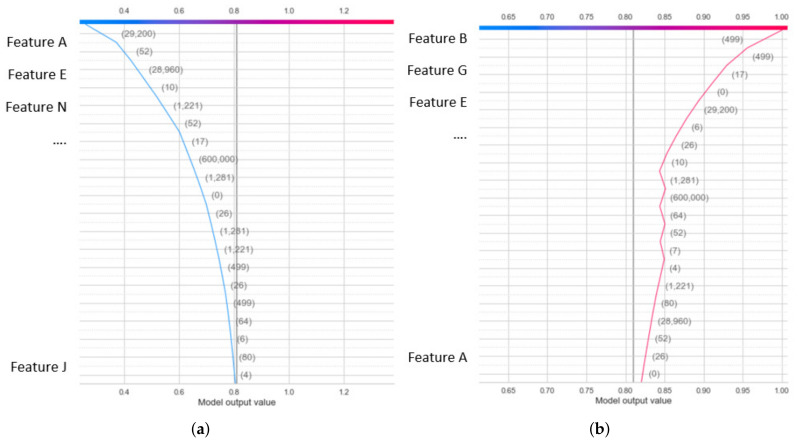
Examples of decision plot. (**a**) Left given the forces of predictors. (**b**) Right give the forces of predictors.

In XAI, local explanations explain how a model makes decisions. The local explanation using the SHAP values via each individual SHAP value which explains why the ML model gives its decision and the contributions of the predictors/features. A decision plot was used to visualize local explanation results to explain the classifiers’ predictions. A decision plot is a good choice when we need to present many predictors/features of the dataset. Figure 2 shows the decision plot example. In particular, the model output is represented by the *x*-axis. The model features are listed on the *y*-axis. At the top of the plot, each observation’s predicted value corresponds to each line striking the *x*-axis. This value represents the color of the line in a spectrum. From the bottom to the top of the plot, SHAP values for each predictor/feature were added to the model’s base value.

### 3.3. The Proposed Method

**Figure 3:**
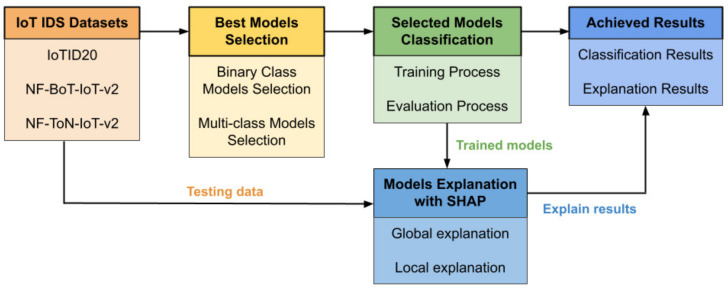
The main concept of the proposed architecture.

This section describes the general proposed architecture. The main concept of the proposed architecture is illustrated in Figure 3. IoT IDS datasets, which are IoTID20, NF-BoT- IoT-v2, and NF-ToN-IoT-v2, have been used for the experiment in the proposed method. The output of the proposed method is the classification results and their explanations. In addition, the three main components of the proposed architecture are the best model selection method, the selected model’s classification, and the model explanation with SHAP.

**Figure 4:**
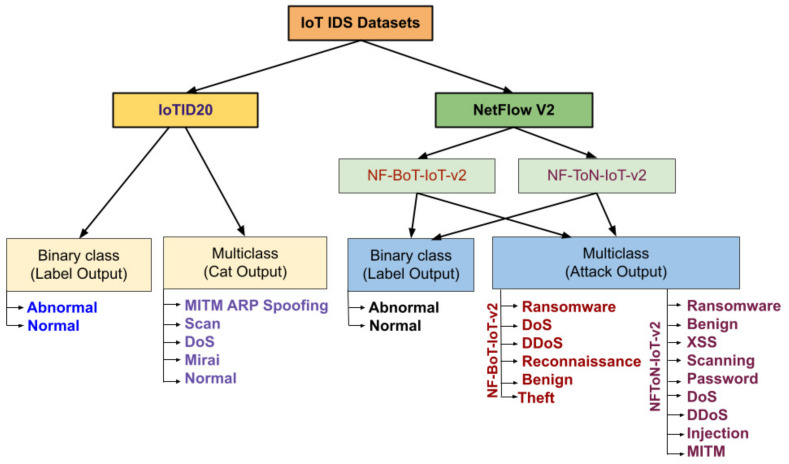
IoT IDS dataset used.

First, for the input data and output results of the proposed method, the related IoT IDS datasets were chosen as input, after which the proposed method can process and obtain the output results. Then, two recent publicly available IDS datasets, including the IoTID20 dataset and NetFlow IoT V2 datasets have been chosen. In the NetFlow IoT V2 datasets, two related IoT IDS datasets have been chosen: NF- BoT-IoT-v2 and NF-ToN-IoT-v2. Each dataset has two types of data: binary-class output and multi-class output (category). Second, the output data were obtained. Two results obtained are presented here, namely: classification results and explanation results. To classify the results, they have been plotted using the ROC curve and validation curve. To explain the results, the proposed results have been plotted by global explanation with a heatmap and local explanation with a decision plot. Figure 4 shows the IoT IDS dataset used in the experiments.

Second, for the best model selection, some ML models have been selected to train on two types of IDS datasets: binary class and multi-class data. Hence, two types of models have been applied on each dataset: binary class classification and multi-class classification. The best model selection algorithm has been developed using Pycaret. Pycaret is an open source code for workflows. The purpose of this library is a low-code ML and an end-to-end model management tool that builds the Python language. In Pycaret, model training, selection steps are very important. This step relates to the training progress and some of the models’ tasks comprise tuning hyperparameters, model evaluation of some ML models. Serveral performance metrics were used to evaluate these ML models, for example, confusion matrix, AUC, and so on. Based on results of the performance models obtained, it allows to select the best model for further use. After this component finishes its operation, the best model for the data can be determined with the best adjustment of the model hyperparameters in Algorithm 1. This algorithm shows the process of the best model comparison and selection with the tuned model hyperparameters.
**Algorithm 1:** The Best Model Selection and Turned Model Hyperparameters**input**: Original Datasets (oD), Input Feature Columns (ifC), Output Feature             Column (ofC) **output**: Best Models Selection (bmS),               Turned Models Hyperparameters (tmP)
1(1) Preprocess datasets (pD)2   pD←Remove_NULL_values(oD)3   pD←Remove_INF_values(pD)4(2) Model comparison and selection5(2.1) Setup the dataset6   grid←Setup_Data(pD[ifC],target=ofC)7(2.2) Evaluate and compare performance models8   bmS←Compare_Models()9(3) Tune models with adjusted hyperparameters10(3.1) Create the best model selected11   model←Create_Model(bmS)12(3.2) Turn the best model to obtained adjusted hyperparameters13   tmP←Tuned_Model(model)14return (bmS,tmP)


Third, for selected model classification, based on the results of the best model selection method, the best classification model with the highest model analysis metric results in AUC, accuracy, recall, precision, F1 have evaluated and selected. The trained models have then been saved for each dataset. Subsequently, the classification model has been evaluated using methods such as ROC curve plotting and validation curve plotting. The training and evaluation processes of the best-selected model classification with tuned hyperparameters are presented in Algorithm 2.
**Algorithm 2:** Training and Evaluation Process of the Best Selected Models with Turned Hyperparameters**input**: Best Model Selected (bmS), Turned Model Hyperparameters (tmP),             Processed Datasets (pD), Input Feature Columns (ifC), Output Feature             Column (oFC) **output**: Trained Model (tM),               ROC Curve (rC),               Validation Curve (vC)
1(1) Get input and output values2   (1.1) Get the input value of dataset3   x←pD(ifC)4   (1.2) Get the output value of dataset5   tC←pD(oFC)6   (1.3) Label encoding the output value7   y←Label_Encoder(tC)8(2) Split processed dataset into training, validating, and testing data9   trD,teD,vaD←Split_Data(x,y)10(3) Train on the best selected model with turned hyperparameters11   (3.1) Build the best selected model with turned hyperparameters12   model←bmS(tmP)13   (3.2) Train the built model above with training and testing data14   tM←model.fit(trD,teD)15(4) Evaluation trained model16   (4.1) ROC curve evaluation for validating data17   rC←ROCAUC(vaD)18   (4.2) Validation curve for validating data19   vC←ValidationCurve(vaD)20return (tM,rc,vC)


Finally, for model explanation with SHAP, the SHAP method has been used to explain why the selected model classification can decide and make classification results. The Shapley value is calculated by evaluating all possible sets of feature values with and without the *i*th feature. To approximate the SHAP estimation for single feature values, Algorithm 3 was used. First, an instance or sample *x*, feature index *i*, and iteration number *K* needs to be selected. A random sample is selected from the data, and a random feature ordering is generated for each iteration. By combining the values of *x* and *r*, two new samples are obtained. The sample $x_{+i}$ is an interesting sample; the number of possible coalitions exponentially increases. Hence, Strumbelj et al. [70] proposed a solution to solve this, but all values in the order after feature *j* are replaced by feature values from sample *r*.
**Algorithm 3:** SHAP value estimation for single feature value**input** Pre-trained model (*f*), example/instance (*x*), feature index (*i*),            data matrix (*X*), number of iterations (*K*) **output**:  SHAP value for the value of the $i^{th}$ feature(*S*)
1(1) For all k=1,…,K:2   Do random instance *r* from the data matrix *X*3   Choose a random permutation *o* of the feature values4   Order instance *x*: $x_{o}=\left(x_{1},\ldots ,x_{i},\ldots x_{p}\right)$5   Order instance *z*: $r_{o}=\left(r_{1},\ldots ,r_{i},\ldots z_{p}\right)$6   Construct two new instances7      With feature:8      $i:x_{+i}=\left(x_{1},\ldots ,x_{i-1},x_{j},r_{i+1},\ldots ,r_{p}\right)$9      Without feature:10      $i:x_{-i}=\left(x_{1},\ldots ,x_{i-1},r_{i+1},\ldots ,r_{p}\right)$11   Compute marginal contribution:12      $C_{i}^{k}=f\left(x_{+i}\right)-f\left(x_{-i}\right)$13   Compute SHAP value as the average:14      $C_{i}\left(x\right)=\frac{1}{K}\sum_{i=1}^{K}C_{i}^{k}$15    $S\leftarrow C_{i}\left(x\right)$16return *S*


   The SHAP results of global and local explanations have been used to interpret the decision classification results of the proposed method. Trained models and testing data have been used for this component. The explanation decisions for the best models for the classification is presented in Algorithm 4.
**Algorithm 4:** Models Explanation with SHAP**input**: Trained Model (tM),             Testing Data (teD) **output**: Global Explanation with Heatmap (geH),               Local Explanation with Decision plot (leD), 1(1) Get SHAP value (*S*) from Algorithm 32      shap←S3(2) Plot global explanation with heatmap4   (2.1) Declare SHAP explanation for trained model5   shap←shap.Explainer(tM)6   (2.2) Get the SHAP value on testing data7   sV←shap(teD)8   (2.3) Get global explanation with heatmap plot9   geH←shap.heatmap(sV)10   (2.4) Get local explanation with decision plot11   leD←shap.decision_plot(sV)12return (geH,leD)

## 4. Experiment

### 4.1. Related IoT IDS Datasets

This section provides the main information of the related IoT-IDS datasets used in the experiments. They are IoTID20 and NetFlow V2 datasets, including NF-BoT-IoT-v2 and NF-ToN-IoT-v2.

#### 4.1.1. Iotid20

An attack is launched because the growth of IoT devices provides a surface and environment for intruders to develop cyber-attacks. The hackers attack the target IoT network resources by exhausting them with malicious activity. Hence, a new dataset was proposed in [47], namely, IoTID20, for a well-designed dataset for IoT networks, and a reference point to determine anomalous activity across the IoT network. This dataset is a new IoT botnet dataset that comprises two advantages: flow-based features and comprehensive networks. This flow-based feature technique was used to analyze and evaluate flow-based IDS. Thus, the IoTID20 dataset will provide a foundation for the development of new IDS techniques in IoT networks. There are 80 network features and three features for output labels, including binary, category, and sub-categories. In this study, we performed an experiment on two label features: binary and category. This is because the number of examples in the training dataset for each subcategory class label was not balanced and contained minority classes. Minority classes are difficult to predict for ML or DL models. Table 1 shows the description of the dataset.

#### 4.1.2. Netflow V2 Datasets

This dataset is version 2, which was created by the NetFlow technique with 43 extended Netflow features [46]. The data comprise two IoT datasets namely: NF- BoT-IoT-v2 and NF-ToN-IoT-v2.

The first dataset, NF-BoT-IoT-v2, which was generated from the original dataset version, namely the BoT-IoT dataset. From the original and available pcap file, the feature data were extracted. Based on the corresponding attack categories, the flows were labeled. The number of samples of the attack is 600,100 with 97.69%, meanwhile, 2.31% of the samples are benign (13,859 samples). There were four attack categories in the dataset. Table 2 lists the NF-BoT-IoT distribution of all flows.

The second dataset is NF-ToN-IoT-v2, which was generated from the original dataset version, namely the ToN-IoT dataset. From the original and available pcap files, the feature data were extracted. Based on the corresponding attack categories, the flows were labeled. The number of samples of attack is 1,379,274 with 80.4%, meanwhile, 19.6% of the samples are benign (270,279 samples). Table 3 lists and defines the distribution of the NF-ToN-IoT dataset.

### 4.2. Experimental Setup and Evaluation Metrics

In this study, two experiments have been performed. In the first experiment, the best model has been evaluated and selected using performance evaluation metrics such as the ROC curve and validation curve. The second experiment is to explain the model-selected decision in global and local explanations. The purpose is to trust the best AI model selected in this approach. Experiments have been conducted on three public IoT-based IDS datasets.

In the preprocessing, we have divided the dataset into 2 parts for training and testing progress with the following ratio 80:20, respectively. To process input for model classifiers, for input variable features with non-numeric values, we have used character-to-numeric conversion techniques using encoding techniques, such as OrdinalEncoder. This is a technique that will encode categorical features as an array of integers. OrdinalEncoder is called from scikit learner with python language.

Two experiments have been conducted in the following programming environment: OS: Windows 10 education, RAM 32 GB, Intel(R) Core(TM) i7-10700K CPU 3.80 GHz 3.79 GHz; and programming language: Python. Several evaluation metrics have been used to measure and verify the proposed method, which are accuracy, area under curve (AUC), and receiver operating characteristics (ROC), Recall, Precision (Prec.), F1.

**Figure 5:**
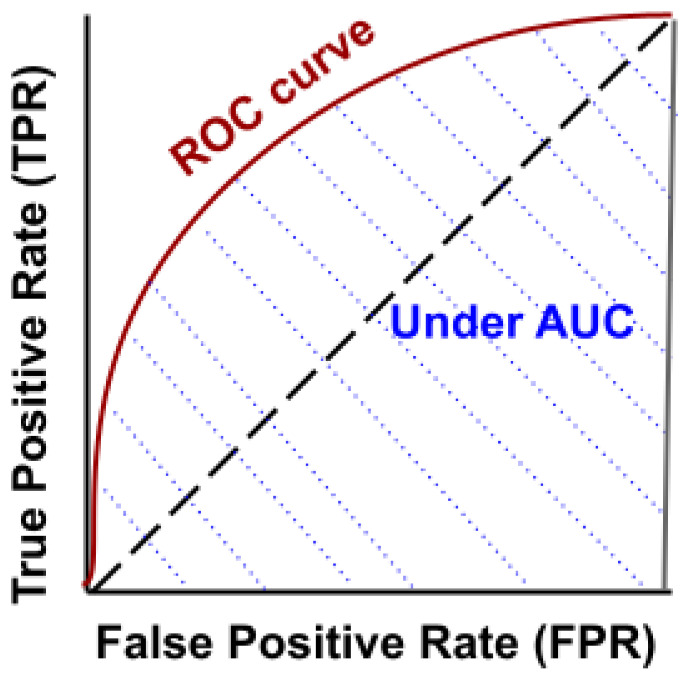
ROC and AUC plotting example.

- *Accuracy*. Accuracy measures how many observations, both positive and negative, were correctly classified.
  (1) $$Accuracy=\frac{TP+TN}{TP+FP+TN+FN}$$
- AUC and ROC. The AUC and ROC curves can be used to measure the performance of the classification models at various threshold settings. The probability curve is presented by ROC, whereas the degree of separability area under the ROC curve is represented by the AUC. These curves show the extent to which the classification model can distinguish between each output class. The higher the AUC, the better the model is for predicting each class correctly. Figure 5 shows the AUC and ROC curves.
  The curve plots two parameters: true positive rate (*TPR* ) and false positive rate (*FPR*), as follows:
  (2) $$TPR=\frac{TP}{TP+FN}$$
  (3) $$FPR=\frac{FP}{FP+TN}$$
- *Recall*. Recall indicates how many of the actual positive cases the model was able to accurately predict.
  (4) $$Recall=\frac{TP}{TP+FN}$$
- *Precision*. Precision indicates how many of the correctly predicted cases actually turned out to be positive.
  (5) $$Precision=\frac{TP}{TP+FP}$$
- *F*1. This combines precision and recall into one metric by calculating the harmonic average between precision and recall.
  (6) $$F1=2\times \frac{Precision\times Recall}{Precision+Recall}$$

### 4.3. Experimental Results

#### 4.3.1. First Experimental Results: Classification Model Performance Evaluation

Two efficient performance evaluation metrics, comprising the ROC curve and validation curve, have been measured to determine the best model classification performance. In the experiment, the results of the classification model evaluation are shown in Table 4.

**Figure 6:**
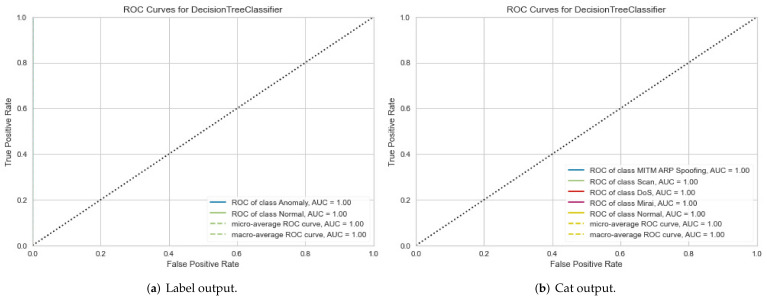
ROC curve results for IoTID20 dataset in two cases: (**a**) Binary class output and (**b**) Multiclass output.

**Figure 7:**
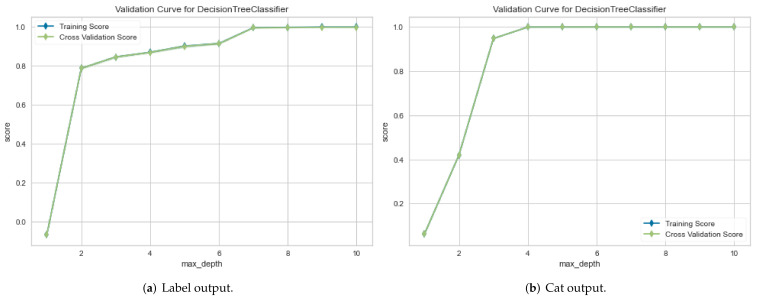
Validation curve results for IoTID20 dataset in two cases: (**a**) Binary class output and (**b**) Multiclass output.

In particular, the details of the evaluation of the two metrics, ROC curve and validation curve for two datasets, are visualized as follows:

For the IoTID20 dataset, in the first metric, the results obtained for the ROC curve in two cases are shown in Figure 6a,b, respectively. In the second metric, the results obtained for the validation curve in two cases are shown in Figure 7a,b, respectively.

**Figure 8:**
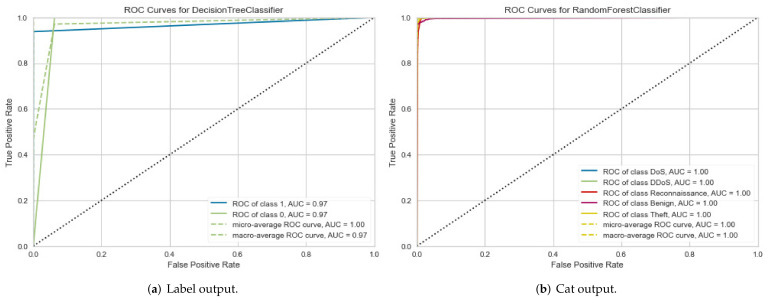
ROC curve results for NF-BoT-IoT-v2 of NetFlow V2 dataset in two cases: (**a**) Binary class output and (**b**) Multiclass output.

**Figure 9:**
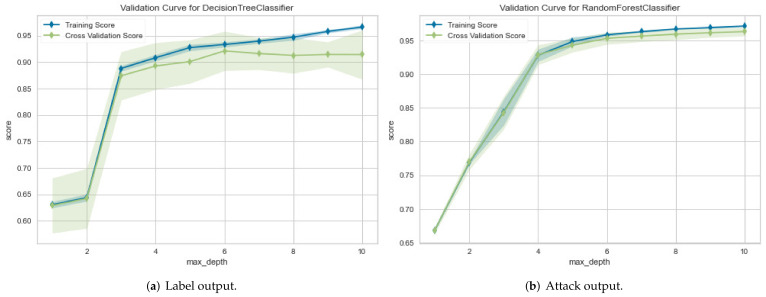
Validation curve results for NF-BoT-IoT-v2 of NetFlow V2 dataset in two cases: (**a**) Binary class output and (**b**) Multiclass output.

For NetFlow version 2 datasets, the first evaluation metric, ROC curve, the result for the NF-BoT-IoT- v2 dataset has been obtained in two cases, as shown in Figure 8a,b, respectively. In the second metric, the results for the validation curve have been obtained in two cases, as shown in Figure 9a,b, respectively. They are similar to those values for the NF-ToN-IoT-v2 dataset.

#### 4.3.2. Second Experimental Results: Explanation of Model Classification Decision

The SHAP value method has been used for global and local interpretation to explain the decisions of the DT and RF classifications on two datasets. In particular, a heatmap representation has been used to explain each model classification on each dataset in global interpretation. In the local interpretation, a decision plot has been employed for the explanation decision of each classification on each dataset.

- Global Explanation with Heatmap

**Figure 10:**
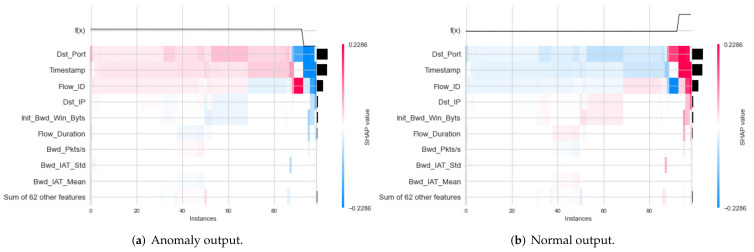
Heatmap curve results of global explanation for IoTID20 dataset in two Label output values: (**a**) 0—anomaly and (**b**) 1—normal.

**Figure 11:**
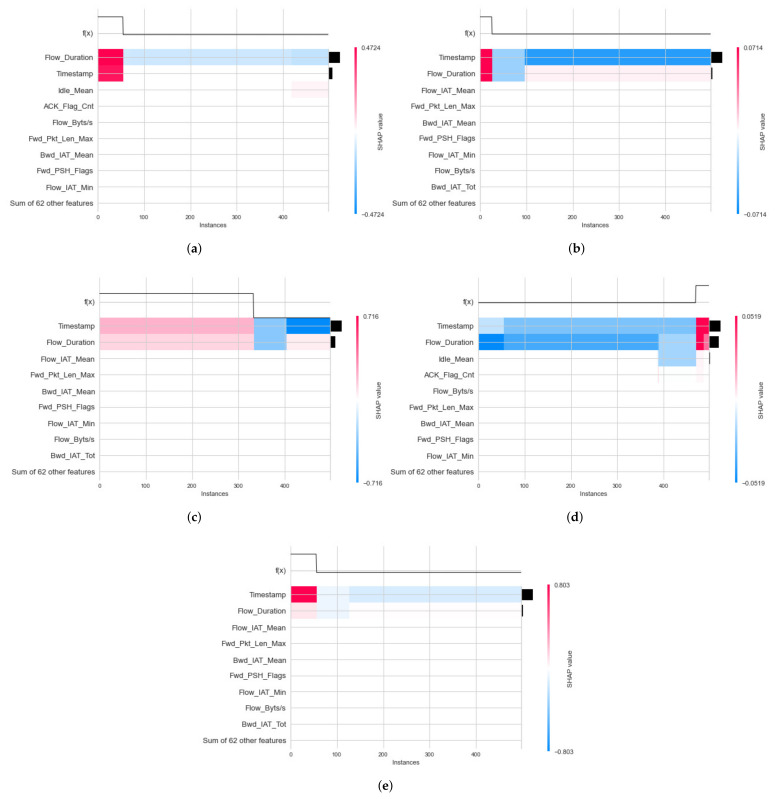
Heatmap curve results of global explanation for IoTID20 dataset in five Cat output values: (**a**) 0—MITM ARP Spoofing; (**b**) 1—Scan; (**c**) 2—DoS; (**d**) 3—Mirai; (**e**) 4—Normal.

For the IoTID20 dataset, the heatmap result for the binary classification of the DT model is presented in Figure 10a,b for two label output values (0—anomaly and 1—normal). In addition, the heatmap results obtained for multiclass classification of the DT model are presented in Figure 11a–e.

**Figure 12:**
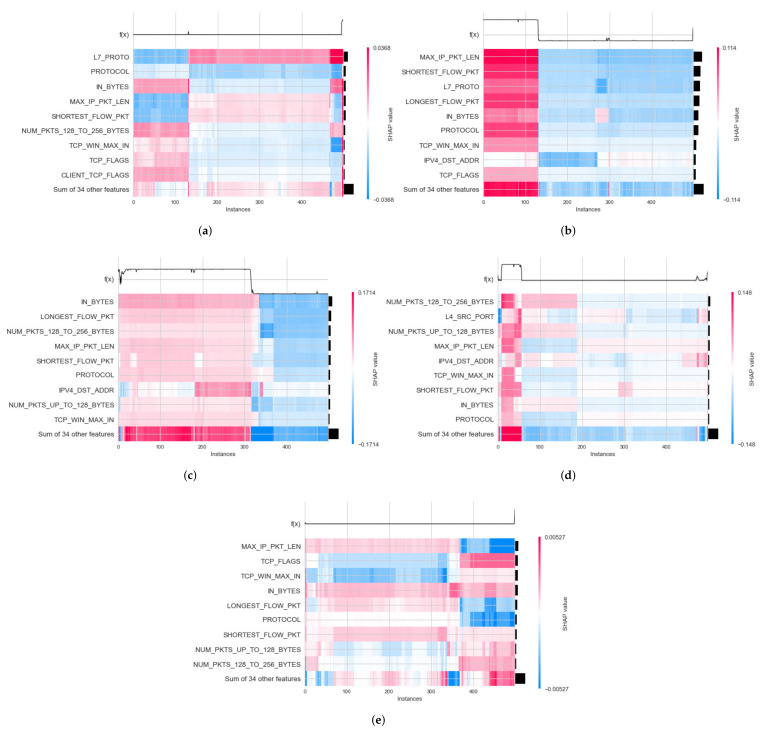
Heatmap results of global explanation for NF-BoT-IoT-v2 of NetFlow V2 dataset in five Cat output values: (**a**) 0—DoS; (**b**) 1—DDoS; (**c**) 2—Reconnaissance; (**d**) 3—Benign; (**e**) 4—Theft.

For the NF-BoT-IoT-v2 dataset, heatmap results for the global explanation of DT and RF models have been obtained in two cases: binary and multiclass classification. In binary classification, the heatmap results of the DT model have been calculated for two label output values (0 and 1). In multiclass classification, the heatmap results of the RF model and the heatmap result of this dataset for multiclass classification are given in Figure 12a–e. Similar to NF-BoT, the IoT-v2 dataset in NF-ToN-IoT-v2, heatmaps for the global results have also been generated for the explanation of the RF model’s decision in two cases: binary classification and multiclass classification.

- Local Explanation with Decision Plot

**Figure 13:**
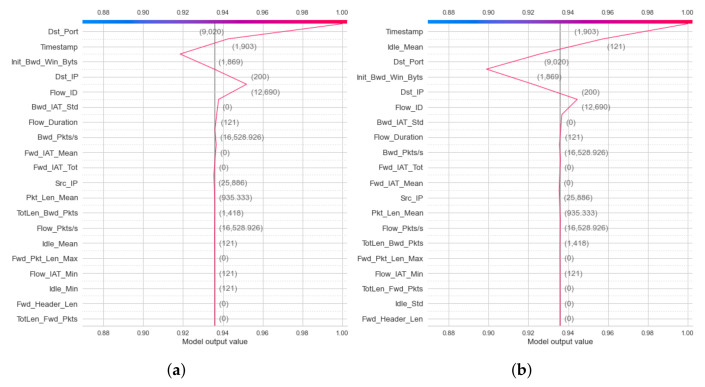
Decision plot results of local explanation for IoTID20 dataset in two Label output values: (**a**) 0—anomaly and (**b**) 1—normal.

**Figure 14:**
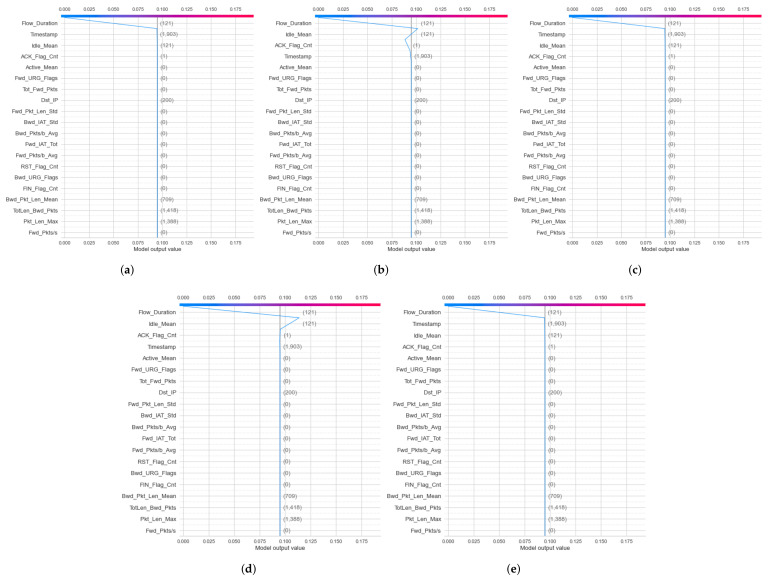
Decision plot results of local explanation for IoTID20 dataset in five Cat output values: (**a**) 0—MITM ARP Spoofing; (**b**) 1—Scan; (**c**) 2—DoS; (**d**) 3—Mirai; (**e**) 4—Normal.

For the IoTID20 dataset, the decision plot result for the binary classification of the decision model has been generated within the local explanation, as shown in Figure 13a,b. In addition, the decision plot results for multiclass classification of the DT model within the local explanation have also been calculated, as shown in Figure 14a–e.

**Figure 15:**
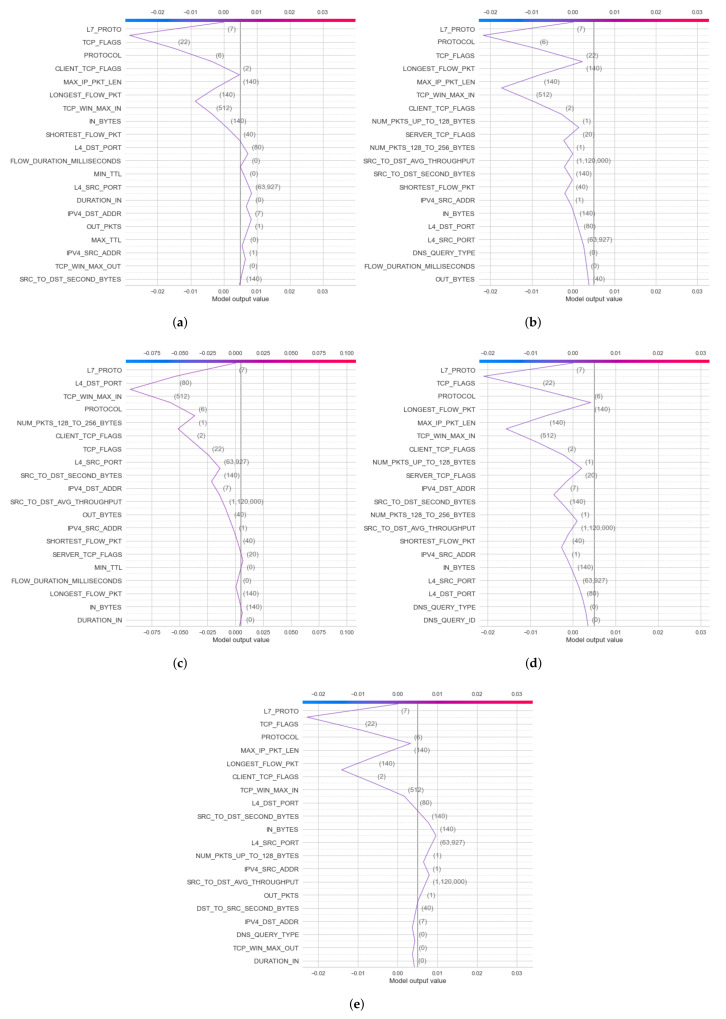
Decision plot results of local explanation for NF-BoT-IoT-v2 dataset in five Cat output values: (**a**) 0—DoS; (**b**) 1—DDoS; (**c**) 2—Reconnaissance; (**d**) 3—Benign; (**e**) 4—Thref.

For NF-BoT-IoT-v2, decision plot results for local explanation of DT and RF models have been calculated in two cases: binary and multiclass classification. In binary classification, the decision plot results of the DT model for two label output values (0 and 1) were obtained. Then, in multiclass classification, the decision plot results of the random forest model have been obtained. The decision plot results of this dataset are presented in the case of multiclass classification in Figure 15a–e. Similar to NF-BoT-IoT-v2 dataset, in NF-ToN-IoT-v2, decision plot results have also been generated for local explanation of random forest model decision in two cases: binary and multiclass classification.

## 5. Discussion and Comparison

In this section, the classification and SHAP explanation results are discussed, and then, the performance accuracy between the proposed method and other IoT-IDS methods is evaluated.

The proposed method can improve the performance detection rate in terms of the AUC and validation metrics. In particular, the performance evaluation of the proposed method is summarized based in Table 4 as follows: for the IoTID20 dataset, the performance evaluation obtained by DT models in both cases, binary class and multiclass classification, are 100% in AUC and validation measurements. In addition, each type of attack has correctly been detected for the two cases. In addition to the NF-BoT-IoT-v2 dataset, the average AUC obtained was 97%, and the validation measurement obtained 97% for binary classification. Meanwhile, the average AUC and validation measurements had 100% and 99% in the multiclass classification case, respectively. With the NF- ToN-IoT-v2 dataset, the average AUC obtained had 100%, and the validation measurement had also 100% for the binary classification. Meanwhile, the average AUC and validation measurements had 93% and 99% in the multiclass classification case, respectively.

Furthermore, the prediction results of the proposed ML methods have been explained by calculating the SHAP values of each feature. Based on the explained results, the key features utilized in the classification prediction have been identified as follows:

First, in the global explanation with heatmap visualization, in the IoTDS20 dataset, the heatmap plot explanation is depicted in Figure 10 for two cases of classifications. Figure 10b shows the low predictions with low SHAP values in f(x) on the right. This means that the DT’s prediction made the decision for this testing sample with normal detection (label output value of 1). In Figure 10b, the heatmap results show high predictions of the DT model (denoted by f(x)) (high values in f(x) on the right) associated with high SHAP values of three important features, including [*Dst_Port, Timestamp, Flow_ID*] (in red color). Similar to multiclass classification for global exploration with a heatmap, five heatmap plots corresponding to five attack types of output were generated, as shown in Figure 11. Among these figures, the highest SHAP values of the important features along with f(x) are as shown in Figure 11d. This figure explains the DT’s detection Mirai attack (Cat output value is Mirai) for this testing sample. In addition, the important features with high SHAP values comprise the timestamp and flow duration for this multiclass classification. Similar to the global explanation of IoTDS20, for the NF-BoT-IoT-v2 dataset, a testing sample was also chosen for a global explanation of the RF model’s prediction by generating heatmap plots for the multiclass classification. The results are shown in Figure 12. The reason why the random forest model predicted DoS attack (with Cat value 0) with the most important feature is [L7_PROTO], as shown in Figure 12a.

Second, in the local explanation with decision plot, the model’s base value is marked by the straight vertical line of the decision plot. Next to the prediction line, feature values are printed for reference. The SHAP values with representative feature effects are accumulated from the base value starting at the bottom plot to arrive at the final score of the top plot. Decision plots are literal representations of SHAP values, making them easy to interpret. In the local explanation with decision plot, in the IoTDS20 dataset, we present the decision plots to explain for DT models’ prediction for two cases classification in Figure 13 and Figure 14. In particular, Figure 13 explains the DT model prediction for each output for the case of binary classification. Besides, Figure 14 shows the DT model prediction for each attack.

Similar to the NF-BoT-IoT-v2 dataset, the result of the decision plot to explain the RF model’s prediction is shown in Figure 15 in case of multiclass classification. Moreover, the vertical format of the decision plot can illustrate the effect clearly for any number of features. For example, Figure 13 and Figure 14 show the 20 most important features out of the total 71 features for IoTDS20 dataset in case binary and multiclass classification. Another example is presented in Figure 14 showing the 20 most important features out of the total 43 features for NF-IoT-BF-v2 dataset in case multiclass classification.

In addition, we have compared the performance accuracy of the proposed method to other methods on the same datasets. The comparison results are shown in Table 5.

Although the DFF and RF methods [68,69] were competitive with the proposed method, our method can be implemented in low-source computing requirements with a CPU and does not require GPU or TPU for complex architecture of DNNs models.

## 6. Conclusions

As a first contribution, this paper proposes a novel and efficient approach to enhance the performance of IoT-IDS systems on three public IoT-based IDS datasets, including the IoTID20, NF-BoT-IoT-v2, and NF-ToN-IoT-v2 datasets. The approach for the ensemble tree models are the DT and RF models. The proposed ensemble tree methods have achieved 100% performance in terms of accuracy and F1 score comparative to other methods on the same datasets used. Although our proposed method with lower AUC measurement results in NF-ToN-IoT-v2 dataset compared to previous DFF and RF methods, the proposed classification results obtained in terms of accuracy and F1 score outperformed the state-of-the-art IoT-IDS methods on the three datasets.

As a second contribution, this paper has approached the SHAP method for both global and local explanations. The global explanation used in the proposed method can interpret the effect of each feature using the heatmap plot technique. In addition, the local explanation used in this framework can interpret the prediction results using the decision plot technique. Both the classification results and explanation results are more useful for enabling cyber-network experts to trust and make better-optimized decisions fast when they face massive IoT-IDS datasets.

In future work, we apply the suitable method to solve the imbalance data issue of the NF-ToN-v2 dataset to improve AUC performance result of our proposed method on this dataset. In addition, we investigate our classification and explanation methods in a practical IoT context to support security experts in their optimized, fast and accurate decision making.

## Figures and Tables

**Table 1:** IoTID20 dataset description.

Label Name	Value	Number of Samples
Label	Normal	40.073
	Anomaly	585.710
Category	Normal	40.073
	DoS	59.391
	Mirai	415.677
	MITM ARP Sppofing	35.377
	Scan	75.265

**Table 2:** NF-BoT-IoT-v2 dataset description.

Label Name	Value	Number of Samples
Label	Normal	13.859
	Anomaly	586.241
Attack	Benign	13.859
	Reconnaissance	470.655
	DDoS	56.844
	DoS	56.833
	Theft	1.909

**Table 3:** NF-ToN-IoT-v2 dataset description.

Label Name	Value	Number of Samples
Label	Normal	270.279
	Anomaly	1.379.274
Attack	Ransomware	142
	Benign	270.279
	XSS	99.944
	Scanning	21.467
	Password	156.299
	DoS	17.717
	DDoS	326.345
	Injection	468.539
	MITM	1.295

**Table 4:** Classification model evaluation on the datasets: IoTID20, NF-BoT-IoT-v2 & NF-ToN-IoT-v2.

Dataset	Best Model	Output Class	Output Label	AUC	Validation
IoTID20	DT	Binary class	Anomaly	1.00	1.00
			Normal	1.00	
			MITM ARP Spoofing	1.00	
			Scan	1.000	
IoTID20	DT	Multiclass	DoS	1.000	1.00
			Mirai	1.000	
			Normal	1.000	
NF-BoT-IoT-v2	DT	Binary class	Anomaly	0.97	0.97
			Normal	0.97	
NF-BoT-IoT-v2	RF	Multiclass	Ransomware	1.00	0.99
			DoS	1.00	
			DDoS	1.00	
			Reconnaissance	1.00	
			Benign	1.00	
			Theft	1.00	
NF-ToN-IoT-v2	RF	Binary class	Anomaly	1.00	1.00
			Normal	1.00	
			Ransomware	1.00	
			Benign	1.00	
			XSS	1.00	
			Scanning	1.00	
NF-ToN-IoT-v2	RF	Multiclass	Password	1.00	0.99
			DoS	1.00	
			DDoS	0.86	
			Injection	1.00	
			MITM	1.00	

**Table 5:** Performance comparison between the proposed method and prior methods on datasets: IoTID20, NF-BoT-IoT-v2 & NF-ToN-IoT-v2.

Dataset	Model	Accuracy	F1	AUC
IoTID20	Ensemble [47]	87%	87%	-
	SLFN [57]	98.42%	98%	-
	CNN-LSTM [58]	98%	98.40%	-
	DT [59]	100%	-	-
	AutoEncoders [60]	94%	-	-
	RF [61]	97.85%	-	-
	Proposed Method	100%	100%	100%
NF-BoT-IoT-v2	Extra trees [45]	93.82%	97%	96.28%
	Extra trees [46]	99.99%	100%	-
	E-GraphSAGE [55]	93.57%	97% -
	RF [68,69]	100%	100%	99.88%
	DFF [68,69]	99.54%	100%	99.96%
	Proposed Method	100%	100%	100%
NF-ToN-IoT-v2	Extra trees [45]	99.66%	100%	99.65%
	Extra trees [46]	98.05%	98%	-
	E-GraphSAGE [55]	99.69%	100%	-
	DFF [56] with CHI	85.61%	91%	85.19%
	RF [56] with COR	99.38 %	100%	99.46%
	RF [68,69]	99.66%	100%	99.61%
	DFF [68,69]	94.74%	96%	98.43%
	Proposed Method	100 %	100%	93%

## Data Availability

Not applicable.

## Abbreviations

The following abbreviations are used in this manuscript:

IDS	Intrusion Detection System
DNN	Deep Neural Network
ML	Machine Learning
DL	Deep Learning
DT	Decision Tree
RF	Random Forest
SHAP	SHapley Additive exPlanation
XAI	eXplanation Artificial Intelligent
DoS	Denial of Service
IoTs	Internet of Things
SDN	Software Defined Networking
ROC	Receiver Operating Computing
SVM	Support Vector Machine
WSN	Wireless Sensor Network
RNN	Recurrent Neural Network
LSTM	Long Short Term Memory
GRU	Gated Recurrent Unit
GPU	Graphic Processing Unit
TPU	Tensor Processing Unit
TCNN	Temporal Convolution Neural Network
H2ID	Hierarchical Hybrid for Intrusion Detection
IG	Information Gain
GR	Gain Ratio
BO-GP	Bayesian Optimization Gaussian Process
DFF	Deep FeedForward
CNN	Convolution Neural Network
LR	Logistic Regression
NB	Naive Bayes
SLFN	Single Hidden FeedForward Neural Network
PSO	Particle Swam Optimization
DBN	Deep Bayesian Network
